# *Plasmodium cynomolgi*: potential emergence of new zoonotic malaria in Southeast Asia

**DOI:** 10.1186/s13071-025-06784-1

**Published:** 2025-04-23

**Authors:** Nantha Kumar Jeyaprakasam, Wei Kit Phang, Shahhaziq Shahari, Indra Vythilingam

**Affiliations:** 1 Biomedical Science Programme, Center for Toxicology and Health Risk Studies, Faculty of Health Sciences, Universiti Kebangsaan Malaysia, Kuala Lumpur, Malaysia; 2 Department of Parasitology, Faculty of Medicine, Universiti Malaya, Kuala Lumpur, Malaysia; 3 Department of Molecular Tropical Medicine and Genetics, Faculty of Tropical Medicine, Mahidol University, Bangkok, Thailand

**Keywords:** *Anopheles*, Diagnostics, Macaques, Malaria, Non-human primate (NHP), *Plasmodium cynomolgi*, Zoonotic

## Abstract

The reported cases of *Plasmodium cynomolgi* in Southeast Asia pose a significant public health concern. Sporadic reports of human *Plasmodium cynomolgi* infections have increased in the past few years, raising attention regarding its potential impact on human populations. Further compounding this issue are the morphological similarities between *P. cynomolgi* and the human malaria parasite *Plasmodium vivax*, which may lead to misdiagnosis and underreporting of *P. cynomolgi* infections. Both in vitro and in vivo studies have shown that *P. cynomolgi* can effectively invade human reticulocytes using mechanisms like those employed by *P. vivax*, underscoring its capacity to infect human hosts if given the opportunity. These studies collectively highlight the parasite's potential to establish infections in humans and emphasize the need for molecular diagnostic tools to accurately detect *P. cynomolgi*. Additionally, challenges in accurate diagnosis and surveillance systems may underestimate the true extent of their impact, making it imperative for healthcare authorities to bolster monitoring efforts and deploy targeted interventions. Strengthening surveillance, improving diagnostic capabilities, and developing targeted vector control strategies are crucial to mitigating the risk of *P. cynomolgi* becoming a major zoonotic disease like its counterpart, *Plasmodium knowlesi*. Thus, this review aims to highlight the current understanding of *P. cynomolgi* infections in human, vector, and macaque hosts based on collated data from previous studies while underscoring the urgent need for enhanced surveillance, accurate diagnostic tools, and effective vector control strategies to mitigate its potential as a significant zoonotic threat in Southeast Asia.

**Graphical abstract:**

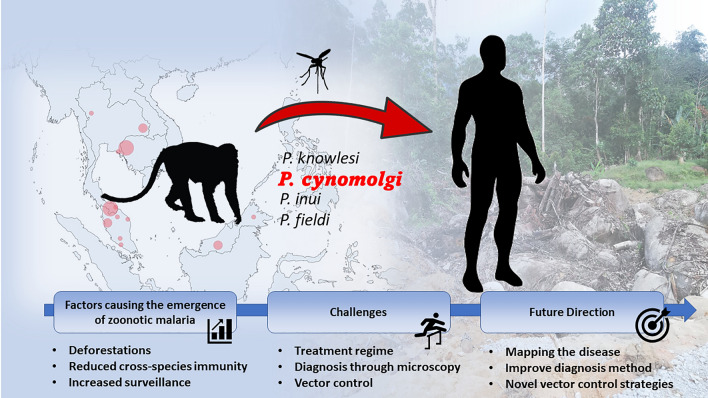

## Background

For the past decades, cases of non-human primate (NHP) malaria caused by *Plasmodium knowlesi* have steadily increased in Southeast Asia [1]. The presence of suitable macaque hosts and competent vectors in this region presents an ideal situation for transmitting zoonotic malaria. Indeed, the overlap of human and macaque habitats, coupled with changes in land use, might have contributed to the rising incidence of NHP malaria [2]. Besides knowlesi malaria, reports on other zoonotic *Plasmodium* such as *Plasmodium cynomolgi* [3–9], *Plasmodium inui* [7, 10–12], and *Plasmodium fieldi* [10] in humans have added another dimension of complexity to malaria elimination in Southeast Asia.

*Plasmodium cynomolgi* is a malaria parasite that primarily infects macaques. In fact, *P. cynomolgi* was the first NHP parasite species to infect humans in the laboratory [13]. The rising incidence of zoonotic malaria caused by *P. cynomolgi* may also be influenced by anthropogenic factors such as deforestation and urbanization [14]. These human activities are believed to increase the prevalence of zoonotic malaria among human populations. Clearing forests for agricultural expansion, logging, or urban development, disrupts the natural habitats of macaques, forcing them to forage for food near forest fringes and nearby villages [15]. This brings humans closer to the macaques, increasing the likelihood of zoonotic malaria transmission when suitable and competent vectors are present [16]. This has been demonstrated in recent studies where *P. cynomolgi* and *P. inui* were the predominant malaria parasites found in macaques and mosquito vectors [1, 17]. Thus, the loss of forest cover directly affects ecosystems and might indirectly contribute to the spread of zoonotic malaria, posing significant public health risks [18]. Additionally, deforestation can alter local microclimates, creating conditions favorable for mosquito proliferation [19]. Therefore, these mosquitoes find more breeding sites in the newly created environments, leading to higher population densities and increased transmission of NHP malaria parasites to humans [19].

Moreover, exploring the zoonotic potential of *P. cynomolgi* through in vitro and in vivo studies reveals intriguing insights into its ability to infect humans [20–22]. Past research has demonstrated the capacity of this malaria parasite species to successfully adapt and proliferate within human hosts, highlighting its potential to cross the species barrier from non-human primates [23, 24]. In vitro experiments have elucidated key molecular interactions between the parasite and human cells, shedding light on the mechanisms underlying its infectivity [25]. Furthermore, in vivo studies involving animal models have provided valuable data on the pathogenesis and transmission dynamics of *P. cynomolgi* in human-like physiological environments [26].

Indeed, the increasing incidence of *P. cynomolgi* cases in humans [3–9] underscores the importance of this new emerging NHP *Plasmodium* as the potential next major zoonotic disease in Southeast Asia, posing a significant public health risk [27]. Furthermore, *P. cynomolgi* in the macaque host produces hypnozoites, a latent stage of the parasite found in the liver, which is responsible for relapses, similarly found in *P. vivax* [28, 29]. Although it is currently unclear whether *P. cynomolgi* relapse occurs in humans, the potential occurrence of hypnozoites is of clinical significance as the treatment for relapsing malaria such as *P. vivax* requires using 8-aminoquinolines drugs (e.g. primaquine), which have a hemolytic effect on glucose-6-phosphate dehydrogenase (G6PD)-deficient individuals [30]. Thus, in this review, we highlight the current collated data on *P. cynomolgi* infections in human, mosquito, and macaque hosts and emphasize the urgent need for improved surveillance, precise diagnostic methods, and effective vector control strategies to reduce its potential emergence as a zoonotic threat in Southeast Asia.

## Methods

For this narrative review article, an extensive literature search was carried out using multiple search engines such as PubMed, Scopus, Google Scholar, Web of Science, and other relevant sites to find information related to *P. cynomolgi*. Articles published between 1960 to 31 May 2024 were screened using the following terms either singly or in combination using Boolean operators (AND, OR): *Anopheles*, control, infection, intervention, in vivo, in vitro, macaques, malaria, mosquitoes, non-human primates, prevalence, primate, *Plasmodium cynomolgi*, simian, vector, and zoonotic. A total of 2744 articles were retrieved from Web of Science, 1879 from PubMed, and 847 from Scopus. Articles were then selected to be included in this review based on their relevance to the topics of prevalence, biology, experimental studies, treatment, diagnosis, and potential control strategies applicable to *P. cynomolgi*. After screening and selection, 79 articles were included in this narrative review. Additionally, a separate search on Google Scholar for publications related to the subject matter yielded 27 more articles, which were also included. Articles that were from informal sources and non-peer/examiner reviewed were excluded from this review.

## Prevalence of *P. cynomolgi* in different hosts

**Fig. 1:**
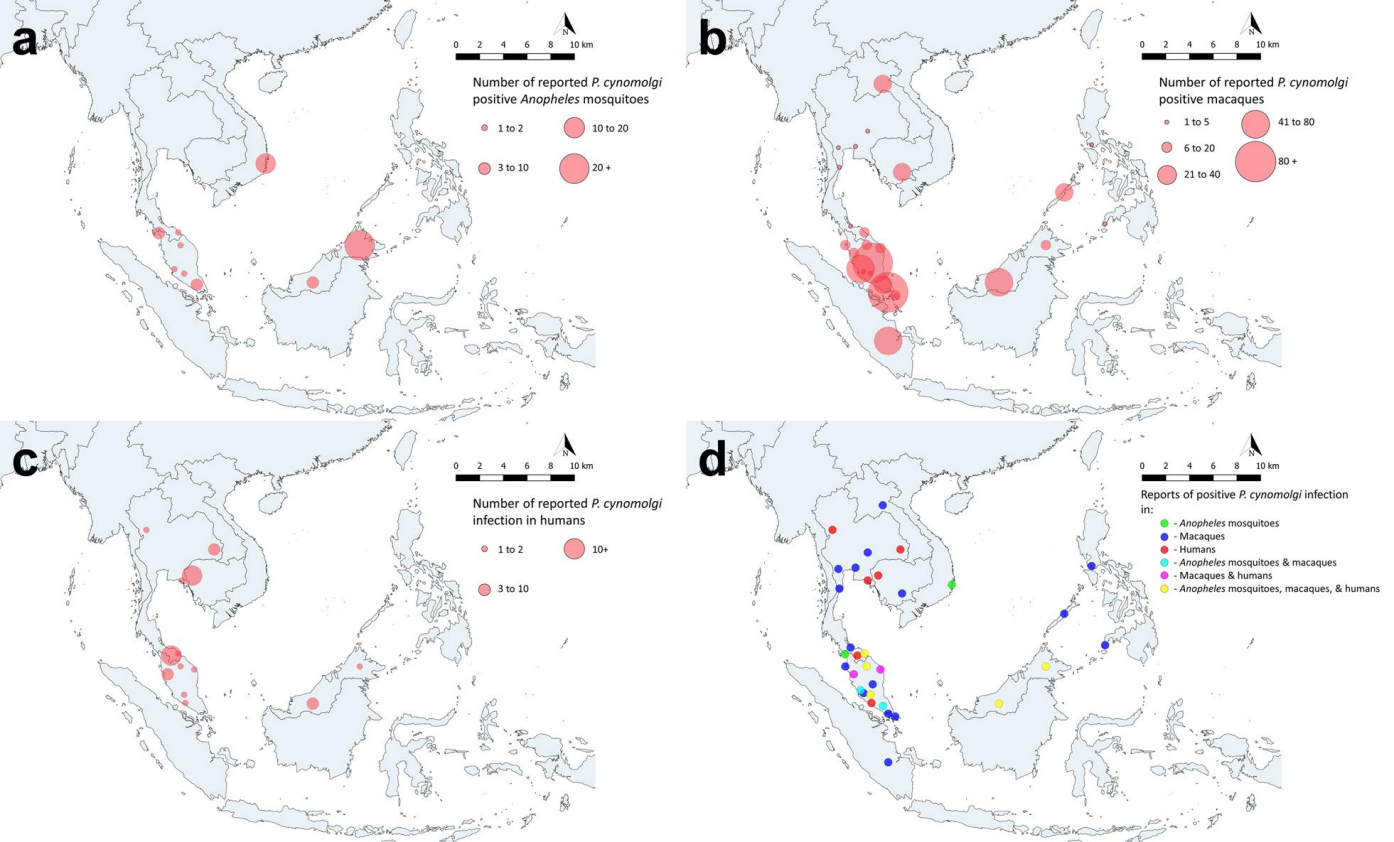
Maps of reported *Plasmodium cynomolgi* infections in *Anopheles* mosquitoes (**a**), macaques host (**b**), and humans (**c**) by first-level administrative divisions. The panel (**d**) shows the overlapping distribution of the vector, macaque host, and past human cases of *P. cynomolgi* infection. Note that only natural *P. cynomolgi* infections in humans were plotted on this map, whereas accidental and experimental infections were excluded. The centroids of each country’s first-level administrative division (state/province) were used to plot the locations of reported infections, except for Singapore and Laos. The author created the Southeast Asian map using QGIS software version 3.6.3 with a base map shapefile modified from the original source (https://gadm.org/data.html)

The distribution of the vector, macaques, and human cases of *P. cynomolgi* is confined to Southeast Asia, where the natural habitats of the *Anopheles* mosquito vector and the macaque hosts overlap with areas of human habitation (Fig. 1). Geographical distribution of the natural reservoir hosts and bionomics of competent vectors are crucial for the successful transmission of this zoonotic malaria [23].

### Vectors

The competent vectors of NHP *Plasmodium* in Southeast Asia are mainly *Anopheles* mosquitoes from the Leucosphyrus Group, which are widely found among various geographic ranges in Southeast Asia [31]. Many species in this group, such as *Anopheles balabacensis*, *An. dirus*, and *An. latens*, are also highly efficient vectors of human malaria [31, 32]. This incrimination of the *Anopheles* Leucosphyrus Group as vectors of *P knowlesi* and other NHP malaria is further substantiated by blood meal analysis, which confirmed the presence of both human and monkey blood in these mosquitoes [33]. Nonetheless, recent studies have also documented the ability of *Anopheles* mosquitoes from the Umbrosus (*Anopheles collessi* and *An. roperi*) and Barbirostris Groups (*Anopheles donaldi*) to transmit zoonotic malaria caused by *P. knowlesi* [34, 35]. However, these need more confirmatory studies as sporozoites were not observed in these mosquitoes and the studies were based only on molecular techniques. Regarding the prevalence of NHP *Plasmodium* identified from the vectors, both *Plasmodium inui* and *P. cynomolgi* showed the highest percentage, followed by *P. knowlesi* (Table 1). Thus, the presence of competent *Anopheles* mosquitoes, particularly from the Leucosphyrus Group, in both peri-domestic and forested areas increases the risk of zoonotic malaria transmission, especially when infected macaque hosts are in the proximity [36]. However, most *Anopheles* mosquitoes infected with *P. cynomolgi* have been documented in Malaysia, likely because of the high numbers of entomological studies conducted in the country. Notably, out of 16 studies conducted across four Southeast Asian countries, 81.3% (13/16) were based in Malaysia.

**Table 1:** Prevalence of *Plasmodium cynomolgi* parasite in *Anopheles* mosquitoes in Southeast Asia

Study period	Country	Location	*Anopheles* group	*Anopheles species* positive for *P. cy*	Total screened	*P. cynomolgi* positive	Mono-infection	Mixed infection	Proportion of *P. cynomolgi* positive *Anopheles*	Screening method	References^b^
1963–1963	Malaysia	Selangor	Leucosphyrus	*An. introlatus*	1	1	1	0	–	SG, innoculation	[37]
1964–1965	Malaysia	Perlis	Leucosphyrus	*An. balabacensis*	42	3	3	0	0.071	SG, innoculation	[38]
2005–2006	Malaysia	Sarawak	Leucosphyrus	*–*	1094	0	0	0	0	MG, SG, Dissection^a^ + PCR	[39]
			Barbirostris	*–*	941	0	0	0	0		
			Umbrosus	*–*	3	0	0	0	0		
			Other group	*–*	466	0	0	0	0		
2010–2013	Vietnam	Khanh Hoa	Leucosphyrus	*An. dirus*	6071	11	6	5	0.002	MG, SG, Dissection^a^ + PCR	[40]
2013–2014	Malaysia	Sabah	Leucosphyrus	*An. balabacensis*	1425	8	4	4	0.004	PCR	[41, 42]
			Non-Leucosphyrus	–	161	0	0	0	0		
2013–2014	Malaysia	Sabah	Leucosphyrus	–	198	0	0	0	0	PCR	[43]
			Barbirostris	–	21	0	0	0	0		
			Umbrosus	–	6	0	0	0	0		
			Other group	–	359	0	0	0	0		
2013–2014	Malaysia	Sabah	Leucosphyrus	*An. balabacensis*	1482	24	3	21	0.016	MG, SG, Dissection^a^ + PCR	[44, 45]
2014–2015	Malaysia	Sarawak	Leucosphyrus	*An. balabacensis*	30	2	0	2	0.067	PCR	[46]
			Barbirostris	–	18	0	0	0	0		
			Not specified	–	17	0	0	0	0		
2014–2014	Malaysia	Sabah	Leucosphyrus	*An. balabacensis*	641	5	3	2	0.008	PCR	[47]
			Barbirostris	–	71	0	0	0	0		
			Umbrosus	–	3	0	0	0	0		
			Other group	–	78	0	0	0	0		
2015–2016	Malaysia	Sarawak	Leucosphyrus	*An. latens*	47	2	0	2	0.043	PCR	[35]
			Barbirostris	–	5	0	0	0	0		
			Umbrosus	*An. roperi*	108	1	0	1	0.009		
			Other group	–	9	0	0	0	0		
2015–2016	Malaysia	Sabah	Leucosphyrus	*An. balabacensis*	254	1	1	0	0.004	PCR	[48]
			Barbirostris	*An. donaldi*	554	1	1	0	0.002		
			Umbrossus	–	2	0	0	0	0		
			Other group	–	259	0	0	0	0		
2015–2015	Philippines	Palawan	Leucosphyrus	–	55	0	0	0	0	PCR	[49]
			Barbirostris	–	1	0	0	0	0		
			Other group	–	67	0	0	0	0		
2018–2019	Thailand	Narathiwat	Leucosphyrus	*An. introlatus*	104	1	1	0	0.010	SG, PCR	[50]
			Barbirostris	–	115	0	0	0	0		
			Umbrosus	*–*	4	0	0	0	0		
			Other group		144	0	0	0	0		
2019–2022	Malaysia	Johor	Leucosphyrus	*An. introlatus*	384	7	2	5	0.018	MG, SG, Dissection^a^ + PCR	[17]
			Barbirostris	–	6	0	0	0	0		
			Umbrosus	–	216	0	0	0	0		
			Other group	–	214	0	0	0	0		
		Kedah	Other group	–	32	0	0	0	0		
		Kelantan	Leucosphyrus	*An. latens*	18	1	0	1	0.056		
			Other group	–	22	0	0	0	0		
		Negeri Sembilan	Leucosphyrus	*An. introlatus*	7	1	0	0	0.143		
			Barbirostris	–	1	0	0	0	0		
			Other group	–	150	0	0	0	0		
		Pahang	Leucosphyrus		55	0	0	0	0		
			Barbirostris	–	7	0	0	0	0		
			Other group	–	42	0	0	0	0		
		Perak	Leucosphyrus	–	10	0	0	0	0		
			Barbirostris	–	4	0	0	0	0		
			Other group	–	100	0	0	0	0		
		Selangor	Other group	–	384	0	0	0	0		

MG: midgut screening; SG: salivary gland screening; PCR: PCR screening

^a^Only studies that used dissected, isolated salivary glands and midguts were described as “dissection”

^b^Only studies that conducted screening for *Plasmodium cynomolgi* detection were included

### Macaques

The most studied hosts for the NHP malaria parasites in Southeast Asia are the long-tailed (*Macaca fascicularis*) and pig-tailed (*Macaca nemestrina*) macaques, which are commonly found near forest fringes and forested areas [51]. Deforestation has driven macaques closer to human settlements in search of food. This increased proximity raises the chances of direct contact between macaques and humans. However, the transmission of *Plasmodium* from NHPs to humans only becomes a risk when suitable mosquito vectors, which are essential for the transmission process, are present [32]. Several studies have reported that *P. inui* has the highest prevalence in macaques, followed by *P. cynomolgi* and *P. knowlesi*, making *P. cynomolgi* the second highest *Plasmodium* species identified in macaques (Table 2). Malaysia has reported the highest number of macaques positive for *P. cynomolgi* in Southeast Asia, partly due to the extensive number of studies conducted there. Across 17 studies from 8 countries in the Southeast Asia region, 47.1% (8/17) of the studies were from Malaysia.

**Table 2:** Prevalence of the *Plasmodium cynomolgi* parasite in macaques in Southeast Asia

Country	Location	Sampling period	Monkey species sampled	Type of monkey	Total monkeys sampled	*P. cynomolgi* positive samples	Proportion of *P. cynomolgi* positive macaques	References
Peninsular Malaysia	Hulu Selangor, Selangor	2014	*Macaca fascicularis*	Wild	70	18	0.257	[52]
Peninsular Malaysia	Pahang	2016	*M. fascicularis*	Wild	34	17	0.500	[53]
		2016	*M. nemestrina*	Wild	5	1	0.200	
	Perak	2016	*M. fascicularis*	Wild	26	16	0.615	
	Johor	2016	*M. fascicularis*	Wild	38	8	0.211	
Peninsular Malaysia	Peninsular Malaysia (Selangor, Negeri Sembilan, Pahang, Perak, Kelantan, Penang)^b^	2010–2014	*M. fascicularis*	Wild	283	79^a^	0.279	[54]
Peninsular Malaysia	Kedah	2016–2019	*M. fascicularis*	Wild	59	0	0	[55]
	Kelantan	2016–2019	*M. fascicularis*	Wild	22	0	0	
	Terengganu	2016–2019	*M. fascicularis*	Wild	58	0	0	
	Pahang	2016–2019	*M. fascicularis*	Wild	188	111	0.590	
	Selangor	2016–2019	*M. fascicularis*	Wild	56	0	0	
	Putrajaya	2016–2019	*M. fascicularis*	Wild	2	0	0	
	Kuala Lumpur	2016–2019	*M. fascicularis*	Wild	30	2	0.067	
Peninsular Malaysia	Johor	2019–2020	*M. fascicularis*	Wild	79	23	0.291	[56]
	Selangor	2019–2022	*M. fascicularis*	Wild	63	22	0.349	
	Pahang	2019–2022	*M. fascicularis*	Wild	73	51	0.699	
	Melaka	2019	*M. fascicularis*	Wild	5	0	0	
	Kelantan	2020	*M. fascicularis*	Wild	31	10	0.323	
	Kedah	2020	*M. fascicularis*	Wild	18	0	0	
	Perak	2020–2022	*M. fascicularis*	Wild	35	1	0.029	
	Perlis	2021	*M. fascicularis*	Wild	6	0	0	
	Negeri Sembilan	2021	*M. fascicularis*	Wild	19	1	0.053	
	Terengganu	2022	*M. fascicularis*	Wild	59	20	0.339	
	Kuala Lumpur	2022	*M. fascicularis*	Wild	19	0	0	
	Putrajaya	2022	*M. fascicularis*	Wild	3	0	0	
Malaysia Borneo	Kapit Division, Sarawak	2004–2008	*M. fascicularis*	Wild	82	52	0.634	[57]
		2004–2008	*M. nemestrina*	Wild	26	9	0.346	
Malaysia Borneo	Sepilok Orangutan Rehabilitation Centre, Sabah	2010–2011	*M. fascicularis*	Wild	26	3	0.115	[58]
		2010–2011	*M. nemestrina*	Wild	15	1	0.067	
Malaysia Borneo	Sarawak	2007–2008, 2012	*M. fascicularis*	Wild	19	9	0.474	[51]
		2003–2012	*M. fascicularis*	Captive	26	1	0.038	
		2007–2008	*M. nemestrina*	Wild	2	1	0.500	
		2003–2012	*M. nemestrina*	Captive	26	3	0.115	
Malaysia Borneo	Ranau, Sabah	2016–2019	*M. fascicularis*	Wild	4	3	0.750	[55]
Thailand	Chacheongsao Province	2017–2019	*M. fascicularis*	Captive	32	2	0.063	[59]
	Ranong Province	2017–2019	*M. fascicularis*	Wild	4	0	0	
	Prachuap Kiri Khan Province	2017–2019	*M. arctoides*	Wild	32	5	0.156	
	Nakornratchasima Province	2017–2019	*M. leonina*	Wild	25	1	0.040	
Thailand	Pattalung^c^	2008–2009	*M. nemestrina*	Wild	13	0	0	[60]
		2008–2009	*M. arctoides*	Wild	4	0	0	
	Pattani^c^	2008–2009	*M. nemestrina*	Wild	1	0	0	
		2008–2009	*M. fascicularis*	Wild	1	0	0	
	Yala^c^	2008–2009	*M. nemestrina*	Wild	62	0	0	
		2008–2009	*M. fascicularis*	Wild	8	0	0	
	Narathiwat^c^	2008–2009	*M. nemestrina*	Wild	373	5	0.013	
		2008–2009	*M. fascicularis*	Wild	186	1	0.005	
		2008–2009	*Semnopithecus obscurus*	Wild	7	0	0	
Thailand	Ranong Province	2006	*M. fascicularis*	Wild	21	0	0	[61]
	Prachuap Kiri Khan Province	2006	*M. fascicularis*	Peri-domestic	78	0	0	
Thailand	Chonburi	2018	*M. fascicularis*	Wild	42	0	0	[62]
	Krabi	2019, 2021	*M. fascicularis*	Wild	44	0	0	
	Lopburi	2018	*M. fascicularis*	Wild	102	0	0	
	Nakhon Si Thammarat	2020	*M. fascicularis*	Wild	25	0	0	
	Narathiwat	2020	*M. fascicularis*	Wild	40	2	0.050	
	Phatthalung	2019–2020	*M. fascicularis*	Wild	85	0	0	
	Phuket	2020	*M. fascicularis*	Wild	30	0	0	
			*M. nemestrina*	Wild	20	0	0	
	Ratchaburi	2018	*M. fascicularis*	Wild	59	1	0.017	
	Songkhla	2018, 2020–2021	*M. fascicularis*	Wild	108	3	0.028	
Singapore	Military protected zone in Western Catchment Area	2007–2011	*M. fascicularis*	Wild	92	40	0.435	[63]
	Peridomestic from various parts of Singapore	2007–2011	*M. fascicularis*	Peri-domestic	65	0	0	
Singapore	Military protected zone in Western Catchment Area	2009–2017	*M. fascicularis*	Wild	379	219	0.578	[64]
	Peridomestic from various parts of Singapore	2008–2017	*M. fascicularis*	Peri-domestic	660	0	0	
Singapore	Singapore (unspecified)	2007	*M. fascicularis*	Wild	40	26	0.650	[65]
Philippines	Puerto Princesa Subterranean River National Park, Palawan	2017	*M. fascicularis*	Wild	40	23	0.575	[66]
	Palawan Wildlife Rescue and Research Center, Palawan	2017	*M. fascicularis*	Captive	25	0	0	
	National Wildlife and Research Centre, Diliman, Quezon City, Manila	2017	*M. fascicularis*	Captive	30	0	0	
Philippines	Zamboanga, Southern Philippines	2012	*M. fascicularis*	Wild	40	1	0.025	[65]
	Batangas, Northern Philippines	2012	*M. fascicularis*	Wild	28	3	0.107	
Taiwan	Chia-shan area Kao-hsiung City, Sourthen Taiwan	2006–2008	*Macaca cyclopis*	Wild	51	0	0	[67]
	Southern Taiwan	2006–2008	*Macaca cyclopis*	Captive	235	0	0	
Indonesia	Southern Sumatra	2010	*M. fascicularis*	Wild	50	48	0.960	[65]
	Bintan Island (Island near Singapore)	2007	*M. fascicularis*	Wild	20	13	0.650	
Cambodia	Vanny	2011	*M. fascicularis*	Wild	54	27	0.500	[65]
Laos	Laos (unspecified)	2013	*M. fascicularis*	Wild	44	28	0.636	[65]

^a^Absolute value was not given in the paper

^b^Unable to accurately discern the prevalence in the individual states

^c^Species were identified by cloning PCR fragments and sequencing positive clones. Species-specific PCR was not conducted; therefore, some species may have been missed because of stochastic effects

### Humans

**Fig. 2:**
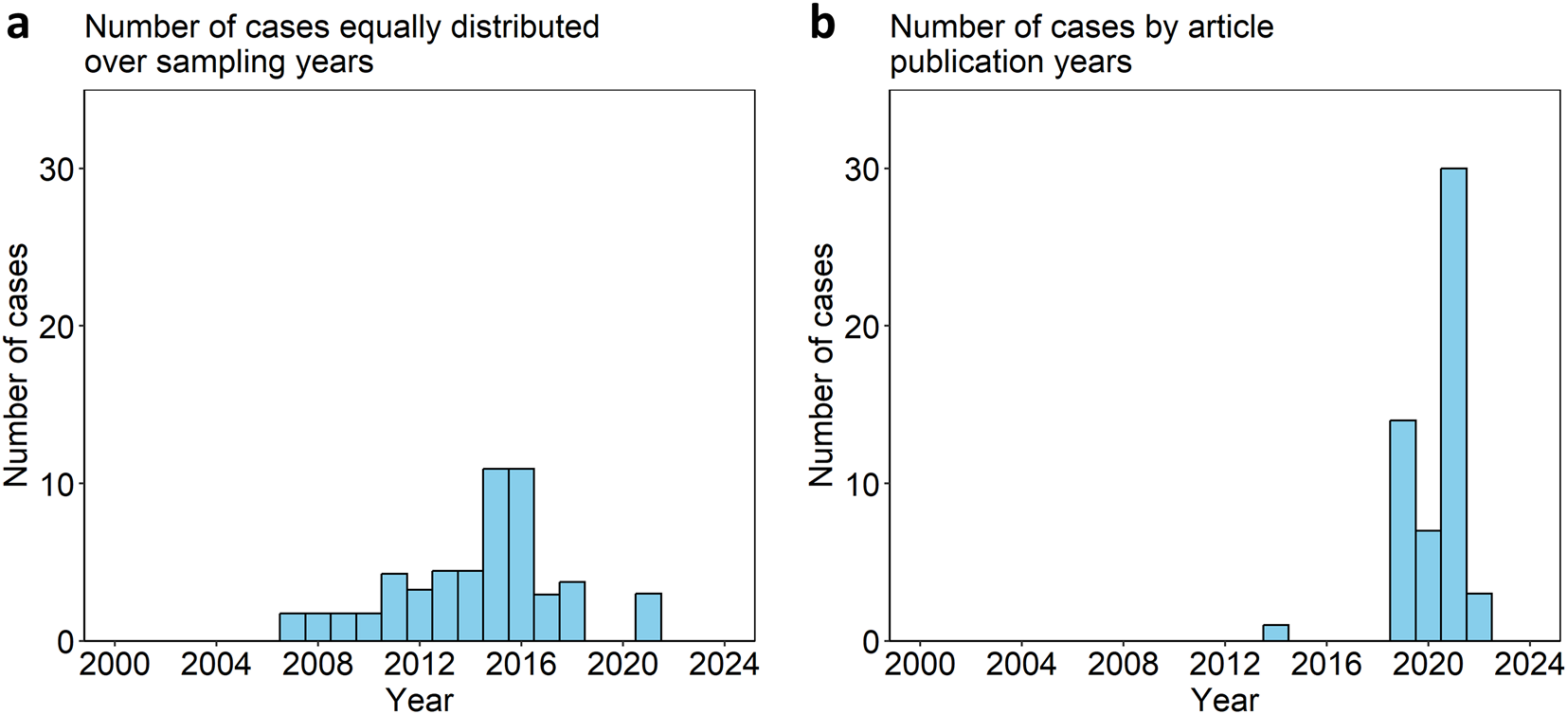
Number of natural *Plasmodium cynomolgi* infections in humans categorized by equal distribution across sampling years (**a**) and by the year of article publication (**b**). Note that data for 2024 may be incomplete as articles were only collected up to 31 May 2024

Although *P. cynomolgi* primarily infects non-human primates, particularly macaques, there has been a growing number of occasional reports of human infections in Southeast Asia (Fig. 2). Besides, some of the cases might be asymptomatic, as evidenced by a study in Malaysia where blood samples collected from aboriginal communities tested positive for multiple NHP malaria parasites, including *P. cynomolgi*, *P. inui*, and others [7]. The reports of these zoonotic infections are relatively infrequent and sporadic compared to knowlesi malaria and other malaria species that infect humans in this region, such as *P. falciparum* and *P. vivax* [68]. Although human cases of *P. cynomolgi* are relatively sporadic (Table 3), they represent an important aspect of malaria epidemiology and highlight the importance of continued surveillance to better understand the prevalence of this zoonotic disease in humans. Although *P. knowlesi* has been reported in many foreign travelers from various countries [1], so far only one case of *P. cynomolgi* has been reported in a Danish traveler who visited Malaysia and Thailand [69].

**Table 3:** Reports of *Plasmodium cynomolgi* cases in human

Study period	Type of study	No. of screened samples	Total no. of *P. cynomolgi* infection	No. of mono-infections	No. of mixed infection	Country	Location	References
NA	Accidental and experimental	–	4	4	0	USA	National Institute of Allergy and Infectious Diseases, National Institutes of Health	[13]
1960	Accidental and experimental	–	3	3	0	USA	The Christ Hospital Institute of Medical Research, Cincinnati, Ohio	[70]
NA	Experimental	–	12	12	0	NA	NA	[71]
NA	Experimental	–	5	5	0	USA	National Institute of Allergy and Infectious Diseases, Bethesda, Maryland	[72]
1962–1963	Experimental	–	1	1	0	Cambodia and USA	Institute for Medical Research, Kuala Lumpur	[73]
NA	Accidental	–	1	1	0	USA	New York University School of Medicine, New York	[74]
NA	Experimental	–	1	0	1	USA	National Institute of Allergy and Infectious Diseases, Bethesda, Maryland	[21]
1977, 1979	Accidental	–	2	2	0	France	Hôspital Pitié-Salpêtrière, Paris	[75]
NA	Accidental	–	1	1	0	NA	NA	[76]
2007 -	Surveillance	144	1	0	1	Thailand	Chanthaburi	[8]^a^
2018		192	1	0	1	Thailand	Narathiwat	
		192	1	0	1	Thailand	Tak	
		239	1	0	1	Thailand	Ubon Ratchathani	
		592	5	0	5	Thailand	Yala	
2007–2018	Surveillance	358	2	0	2	Thailand	Chanthaburi	[10]^a^
		152	1	0	1	Thailand	Narathiwat	
		923	1	0	1	Thailand	Tak	
		639	3	0	3	Thailand	Ubon Ratchathani	
		2123	14	2	12	Thailand	Yala	
2011–2016	Surveillance	55	5	5	0	Malaysia	Perak	[7]
		13	2	2	0	Malaysia	Negeri Sembilan	
		13	1	1	0	Malaysia	Melaka	
		9	1	1	0	Malaysia	Gua Musang, Kelantan	
2011	Case report	-	1	1	0	Malaysia	Hulu Terengganu, Terengganu	[6]
2013–2017	Surveillance	1047	6	0	6	Malaysia	Kapit, Sarawak	[24]
2015–2016	Surveillance	7320	13	11	2	Cambodia	Battambang	[4]
2018	Case report	–	1	1	0	Malaysia	Tanah Merah, Kelantan	[77]
2018	Case report, imported	–	1	1	0	Thailand and Malaysia	Various locations	[69]
2021	Case report	–	3	2	1	Thailand	Yala	[9]

*NA* Data not available

^a^Data may overlap between [8] and [10]

## Emergence of *P. cynomolgi* as a public health challenge

### Reduced cross-species immunity

A possible explanation for the reported *P. cynomolgi* cases in humans is probably the decline in human malaria cases, such as *P. vivax*, which reduces the population’s immunity to *Plasmodium* [78]. Research using in vitro and ex vivo derived *P. cynomolgi* parasites has demonstrated the ability of antibodies generated against *P. vivax* to cross-react and bind to antigens on *P. cynomolgi*, and vice versa, primarily evidenced through Western blot and immunofluorescence assays [79, 80]. However, more recently, it was demonstrated that polyclonal antibodies raised in 2-month-old New Zealand rabbits against recombinant *P. vivax* antigens*, P. vivax* merozoite surface protein 1 (PvMSP1-19), *P. vivax* merozoite surface protein 8 (PvMSP8), and a chimeric protein consisting of a fusion between the two proteins (PvMSP8 + 1) were able to block *P. cynomolgi* invasion in vitro [80]. This shows that antibodies raised against *P. vivax* could have a protective effect against *P. cynomolgi* infections providing evidence to support the cross-species immunity hypothesis.

However, it should be noted that cross-species challenge in in vivo models from previous research showed that patients immunized with *P. cynomolgi* blood stage parasites showed no protection against subsequent *P. vivax* challenges [21]. Unfortunately, to date, there have been no further related in vivo studies in humans, suggesting the need for further study to elucidate the implications of cross-species immunity between humans and NHP *Plasmodium* species. Similarly, a separate study on rhesus macaques (*Macaca mulatta*) immunized against recombinant *P. vivax* MSP1 protein also failed to show protection against subsequent *P. cynomolgi* challenge [79]. However, it was noted that the immunized macaques did show a significant decrease in peak parasitemia and average parasitemia during the observational period. This suggests that although the antibodies raised against *P.* vivax did not protect the macaques from acquiring the infection, the antibodies produced could aid in regulating the infection from becoming severe by controlling the parasitemia. Thus, overall, cross-species immunity does seem to influence the course of an infection. However, to what extent this contributes to the recent rise in *P. cynomolgi* cases remains to be confirmed and could be validated with future in vitro and ex vivo studies.

### Morphological similarities and improved diagnostic methods

The morphological similarities between the NHP *Plasmodium*, *P. cynomolgi*, and human *Plasmodium*, *P. vivax*, might have historically led to underreporting of *P. cynomolgi* infections in humans [81]. Due to their similar appearance under microscopy, *P. cynomolgi* infections have often been misidentified as *P. vivax* [77]. This is especially true because the gold standard for malaria diagnosis in most countries is still observing blood film using a microscope [82]. However, with improved molecular detection methods, more *P. cynomolgi* cases in humans are being reported. Advanced molecular techniques, such as PCR assay, target specific genetic markers that can accurately distinguish *P. cynomolgi* infections from other malaria parasites [83]. Additionally, serological assays capable of detecting antibodies against *P. cynomolgi* antigens can identify past or current infections, even in asymptomatic individuals [84]. By employing these improved diagnostic methods, healthcare professionals can more effectively identify NHP *P. cynomolgi* cases, leading to better understanding, surveillance, and management of this emerging public health concern. With the implementation of this enhanced detection method, it is anticipated that more *P. cynomolgi* cases will soon be identified [1].

### Asymptomatic infections and in vitro/in vivo invasion characteristics

*Plasmodium cynomolgi* infections in humans tend to present as asymptomatic infections with sub-microscopic parasitemia [4, 7, 8]. Thus, the prevalence of *P. cynomolgi* in humans has possibly been underreported. Experimental in vivo infections in the 1970s showed that *P. cynomolgi* infections in humans tend to result in low, self-resolving parasitemia [20, 21]. A previous study using ex vivo experiments showed that the *P. cynomolgi* B/M strain preferentially invades reticulocytes (i.e. young RBCs) when parasites are allowed to invade human RBCs [22]. Furthermore, in *P. vivax* infections, a study has shown that most parasites reside in extravascular erythropoietic tissue (e.g. bone marrow, spleen, and liver), meaning that peripheral blood parasites do not accurately reflect total parasite load [85]. However, this has not been confirmed in human *P. cynomolgi* cases. Thus, the potential sequestration of parasites and *P. cynomolgi*'s preference for reticulocytes in human RBCs may result in low peripheral blood parasitemia, increasing the likelihood of undetected cases in humans [23]. Consequently, traditional diagnostic methods such as microscopy may fail to detect the presence of the parasite when its concentration is below the detection threshold [81].

However, *P. cynomolgi* invasion into human RBCs is currently poorly understood. Past studies have shown that there was no preference for RBC age when *P. cynomolgi* parasites were allowed to invade *M. mulatta* RBCs but a strict preference for reticulocytes in human RBC invasion [22]. This behavior of differential host cell preference could be one of the reasons why the prevalence of *P. cynomolgi* is higher in macaques while relatively lower in humans [22]. However, why *P. cynomolgi* tends to present as an asymptomatic infection in humans is currently unclear, and the differential host cell preference could be one of the contributing factors.

## Implications for public health

### Zoonotic and non-zoonotic potential

*Plasmodium cynomolgi* can cross species boundaries to infect both non-human primates (NHPs) and humans [20]. In some regions in Southeast Asia where humans, NHPs, and *Anopheles* vectors coexist, there is a presumably higher risk of NHP malaria spillover due to the sharing of habitats between these host populations [86]. Nevertheless, only sporadic occurrences of natural *P. cynomolgi* infections in humans have been reported compared to more prevalent zoonotic *P. knowlesi* cases [87].

Despite reports of natural human infections with *P. cynomolgi*, our understanding of its potential for human-to-human transmission is limited. However, experiments in the 1960s showed the possibility of human-to-human transmission [13, 70, 71, 73]. In one study, *P. cynomolgi* M strain and B strain were successfully transmitted from human to human using *Anopheles freeborni* mosquitoes to infect the participants [71]. However, two cases of accidental human infections with *P. cynomolgi* through mosquito bites have been reported in France [75] and in three different laboratories in the US [13, 70, 74]. In all cases, infections were acquired through mosquito bites. Nevertheless, to date, there is no evidence that *P. cynomolgi* can spread from human to human in the natural setting. However, this zoonotic potential raises concerns about new sources of emerging zoonotic malaria infections, emphasizing the need for ongoing research, surveillance, and control measures to prevent potential outbreaks in human populations.

### Occupational safety and public well-being

People working in forests or residing near forest fringes are at heightened risk of NHP malaria infections due to the potential transmission of this parasite by *Anopheles* mosquito vectors inhabiting these areas [87]. The ability of macaques, especially long-tailed macaques, to thrive in human-altered environments has made them the most common NHP species frequently encountered by people in these areas [88]. Thus, the presence of suitable macaque hosts and competent vectors in these areas increases the risk of NHP malaria infection for people living on forest fringes or engaging in forest-related activities [89]. For example, in Thailand, a case report identified three patients infected with *P. cynomolgi*: two worked in agriculture and one in the army, and all had engaged in activities near forested areas [9]. Indeed, *P. cynomolgi* poses a threat to occupational and health security, as infections can lead to complications such as cephalgia, anorexia, myalgia, and nausea [6]. Symptoms such as muscle pain, malaise, fever, headache, abdominal pain, decreased platelet count, thrombocytopenia, chills, and rigor were reported [69]. These symptoms are similar to those caused by other malaria species with uncomplicated symptoms. Additionally, cases of *P. cynomolgi* co-infections were also reported among symptomatic malaria patients in Thailand [8]. Nevertheless, most of the *P. cynomolgi* infections reported in humans to date were asymptomatic or caused only mild symptoms [81]. Unfortunately, the severity of *P. cynomolgi* infection in humans remains to be fully elucidated. However, like *P. vivax*, *P. cynomolgi* can form hypnozoites that can cause relapses, which might complicate treatment and follow-up [77]. Thus far, the presence of hypnozoites and relapse properties of *P. cynomolgi* have only been proven in macaques [28, 29]. In an experimental study, five volunteers were infected with the B strain of *P. cynomolgi*, with one volunteer exhibiting parasitemia for up to 59 days before all volunteers were treated with chloroquine and primaquine [72]. However, to date, only one survey has reported *P. cynomolgi* detected twice in two human individuals, with an intervening interval of approximately 3 months, but whether they were relapses, reinfections, or persistent infections could not be ascertained [4].

## Challenges

### Challenges to malaria diagnosis

Morphologically, microscopic screening of asexual stages of *P. cynomolgi* can be misidentified as *P. vivax* because they are morphologically indistinguishable; therefore, any *P. vivax*-like infections in humans are likely to be characterized as *P. vivax* through microscopy [20]. This parallels the lesson learned from the misdiagnosis of *P. knowlesi*, where samples initially identified as *P. malariae* by microscopic examination were later confirmed to be *P. knowlesi* via PCR [90]. Indeed, the difficulty in distinguishing between *P. vivax* and *P. cynomolgi* has been noted in recent studies [22, 77]. In most instances, *P. cynomolgi* was not initially found as it was not included in the routine microscopic screening for malaria.

### Effectiveness of the current treatment regime

While the treatment for *P. cynomolgi* infections in humans remains poorly studied, available evidence suggests that existing antimalarial drugs effective against *P. vivax*, such as chloroquine and primaquine, will probably be useful in treating *P. cynomolgi* infections. Based on the first natural human case of *P. cynomolgi* infection reported in Terengganu, Malaysia, the patient, initially diagnosed with *P. malariae*/*P. knowlesi*, was treated with chloroquine. The fever and symptoms resolved without complications or relapses [6]. In a case series from Thailand, all *P. cynomolgi*-infected patients recovered quickly and experienced no recurrences over a 3-month follow-up period, indicating the efficacy of *P. vivax* antimalarial regimens using chloroquine and primaquine in treating *P. cynomolgi* [9]. Similarly, a separate case study in Kelantan, Malaysia, recommended using chloroquine for the acute phase of infection and primaquine to target the liver stages of the parasite and prevent relapses, like the treatment of *P. vivax* infections [77]. The combination of chloroquine and primaquine has also been used to treat volunteers who participated in human *P. cynomolgi* infection experiments [21, 72, 73]. Regarding drug resistance investigations, a study conducted in Cambodia found no evidence of drug resistance among *P. cynomolgi*-positive samples based on gene encoding mutation for dihydrofolate reductase-thymidylate synthase (dhfr-ts) [4]. Further research and development of molecular tools are essential to enhance the accuracy of diagnosis and treatment efficacy for *P. cynomolgi* infections in humans.

## Future recommendations

### Mapping the disease

Mapping the disease involves understanding its distribution and prevalence, which is crucial for targeted intervention. Researchers can use geospatial data and epidemiological studies to identify regions where *P. cynomolgi* transmission exists. In Southeast Asia, where cases of NHP malaria caused by *P. cynomolgi* have started to emerge, mapping can help prioritize resources for control efforts. This approach has been successfully applied in identifying areas with a higher risk for knowlesi malaria transmission [87, 91–93]. In Malaysia, the higher number of confirmed *P. cynomolgi* cases can be attributed to intensive entomological research and numerous macaque studies. However, it is important to consider the potential for substantial sampling biases in the available data, which may affect the perceived distribution of the parasite. By modeling the parasite's geographical distributions, researchers can identify areas where *P. cynomolgi* has not yet been reported, guiding public health officials in more strategized epidemiological surveillance in those areas. This approach can aid in early detection and timely responses to outbreaks [92]. Furthermore, it helps understand the transmission dynamics of this emerging zoonotic disease, essential in improving overall public health strategies and reducing the incidence of NHP malaria in Southeast Asia.

### Inclusion of NHP malaria parasites in screening and improving diagnosis methods

Non-human primate malaria parasites might not be recognized in routine malaria surveillance that relies solely on microscopy, mainly because of the morphological similarities between zoonotic and human *Plasmodium* species [20]. Furthermore, many countries only include human *Plasmodium* in their routine screening for malaria-positive blood samples, except for *P. knowlesi* in some countries in Southeast Asia with a higher prevalence of knowlesi malaria [1]. Thus, including *P. cynomolgi* and other NHP parasites in existing routine malaria screening and notification systems can help identify cases and manage outbreaks effectively. This is particularly important for malaria detection in areas where human infection with *P. cynomolgi* has been previously reported or where *P. cynomolgi* infection is known to occur in local NHP populations or vectors.

Investment in improving molecular screening methods to supplement existing microscopy-based diagnostics, such as PCR-based tests, can enhance diagnostic accuracy and reduce misdiagnosis [94]. As the commonly used PCR may be expensive, governments could allocate funds to districts or states that have reported higher incidences of NHP malaria. Targeted fund allocation ensures that resources are directed where they are most needed, maximizing the impact of intervention efforts. This eventually leads to early detection and treatment, which in turn can improve malaria case management in the country.

It is also essential to explore cost-effective alternatives such as loop-mediated isothermal amplification (LAMP), which is simpler, faster, and cheaper while maintaining high sensitivity and specificity [95, 96]. Training local healthcare workers and leveraging existing infrastructure for sample collection and testing can further reduce costs. In addition, developing a sensitive rapid diagnostic test (RDT) to detect *P. cynomolgi* and other NHPs can be very useful for screening communities living in hard-to-reach areas because of its affordability and portability [97]. Rapid diagnostic tests specifically designed for *P. cynomolgi* can improve diagnosis, increasing the frequency and coverage of screening. However, it should be noted that developing RDTs to detect *P. cynomolgi* can present several challenges. This includes difficulties in detecting samples with low parasitemia, which are usually observed in patients infected with *P. cynomolgi* [8, 24], and the possibility of cross-reactivity with other human *Plasmodium* due to genetic similarities [98]. Indeed, overcoming these challenges requires much research and development in producing sensitive and specific RDTs to ensure accurate detection and effective malaria control.

Another potential method for detecting *P. cynomolgi* is antibody detection using enzyme-linked immunosorbent assays (ELISAs) [99]. ELISA is a sensitive and specific immunological technique that detects antibodies produced in response to infection, making it useful for diagnosing past or ongoing infections, even when parasitemia is low or undetectable by microscopy. Nevertheless, while advances in diagnostic technologies have led to detection of more cases, this does not imply that the disease was previously absent from the population. Infections by *P. cynomolgi* may have been unintentionally overlooked as they are often asymptomatic and can easily be indistinguishable from other human malaria parasites because of their similar morphology. Further surveillance involving targeted mass screening of the population, complemented by advanced diagnostics technologies, should be carried out to investigate the dynamics of this zoonotic malaria transmission.

### Provide awareness and health education

As *P. cynomolgi* is an emerging parasite in humans, there may be a lack of knowledge about it among clinicians and the public [77]. Educating communities about NHP malaria and its symptoms, prevention, and treatment is essential to reduce transmission and improve health-seeking behavior. Health authorities can launch public awareness campaigns using various media platforms, including radio, television, and social media [100]. These awareness campaigns can be integrated with existing campaigns on *P. knowlesi* malaria to avoid incurring additional costs while ensuring that both zoonotic malaria species receive appropriate attention. Besides, community health workers can also be trained to educate and engage with the local populations, particularly those working or staying close to forested areas. Additionally, clinicians should be sensitized to NHP malaria caused by *P. cynomolgi* and other species and its treatment for better patient management.

### Research on novel vector control strategies

Besides *P. knowlesi*, the emergence of other NHP *Plasmodium* that can naturally infect humans, such as *P. cynomolgi*, *P. inui*, and *P. fieldi*, in Southeast Asia is worrying as strategies to control the mainly forest-dwelling mosquito vectors are lacking [31]. Traditional vector control methods, such as indoor residual spraying (IRS) and insecticide-treated bed nets (ITNs), might be ineffective against vectors of zoonotic malaria that exhibit exophagic characteristics [101]. Therefore, there is a dire need for more studies focusing on development, evaluation, and implementation of integrated vector management to control the transmission of simian parasites from macaques to humans.

Some possible methods that could be further investigated to control zoonotic malaria vectors include outdoor residual spraying (ORS) [102, 103] and attractive toxic sugar baits (ATSBs) [104, 105]. The risk of acquiring zoonotic malaria can also be reduced by using a repellent to prevent mosquito bites when engaging in outdoor activities. One of the promising methods is DEET (N,N-diethyl-meta-toluamide)-impregnated anklets and wristbands [106]. By incorporating DEET into anklets and wristbands, these devices can provide a localized zone of protection around the wearer. The convenience and ease of use of these wearable repellents' make them a practical addition to other zoonotic malaria prevention strategies.

## Conclusions

*Plasmodium cynomolgi* has the potential to emerge as an important public health challenge in Southeast Asia, necessitating a deeper understanding of its biology, transmission dynamics, and clinical implications. The detection of *P. cynomolgi* infections in humans and the high prevalence of the parasites in both the vectors and macaque host further highlight the possible emergence of *P. cynomolgi* infections in humans. However, the transmission dynamics among macaques, mosquitoes, and humans remain poorly understood, making it difficult to assess the true risk of zoonotic spillover. Nevertheless, past in vitro studies have provided valuable insights into the behavior and characteristics of *P. cynomolgi*, contributing to our understanding of this emerging threat. Indeed, much remains to be explored, and further research is crucial for effectively addressing the challenges it presents and developing targeted interventions for its control and elimination.

## Data Availability

No datasets were generated or analysed during the current study.
